# Supplementary material for: Improving HIV pre-exposure prophylaxis (PrEP) adherence and retention in care: Process evaluation and recommendation development from a nationally implemented PrEP programme
Source: PLoS One. 2023 Oct 9;18(10):e0292289. doi: 10.1371/journal.pone.0292289 (PMC10561843; doi:10.1371/journal.pone.0292289)
Supplement: S1 Table — (DOCX) [file pone.0292289.s001.docx]

**S1 Table. Priority area 1 - A BCW analysis of ‘PrEP providers support PrEP users to adhere to their chosen regimen’**

| **Barriers** | **Facilitators** | **Indicative quotes** | **TDF domains** | **Intervention Functions** | **Potential BCTs**  from the BCTTv1 (Michie et al. 2013) | **Initial recommendations for those considering implementing PrEP at scale**  Numbers in brackets = BCTs | **Post-APEASE and expert input decision**  Accept/Reject/Modify | **Agreed final recommendations** **for those considering implementing PrEP at scale** |
| --- | --- | --- | --- | --- | --- | --- | --- | --- |
| PrEP providers find it difficult to support PrEP users to adhere to their chosen regimen because they have to rely on user-reported adherence which may over-report good adherence due to a desire to please PrEP providers | **--** | “*With PrEP it’s very difficult, you don’t have an objective measure of their adherence so you only know what patients are telling you and generally patients want to please their clinicians so they will say to you, oh, no, I’ve been taking it. So, whether that’s accurate or not I don’t know*.” (Sexual healthcare professional) | Social influences | Environmental restructuring  Enablement | 6.2 Social comparison  12.2 Restructure the social environment  1.2 Problem solving  7.1 Prompts/cues | 39. Sexual healthcare professionals should draw attention to the challenges of taking PrEP based on what they know about the experiences of other PrEP users (6.2) and cultivate a no blame and non-judgemental approach to encourage an open dialogue about any adherence issues or concerns (12.2)  40. Sexual healthcare professionals should engage PrEP users in a discussion of factors that (could) influence adherence and generate suitable solutions (1.2) then document the discussion in the electronic patient record as a useful basis for opening the adherence conversation at the next appointment (7.1) | 39. Reject – part of routine practice not PrEP specific  40. Reject – not sure that the electronic patient record is a good place for opening conversations about adherence | -- |
| PrEP providers find it difficult to support PrEP users to adhere to their chosen regimen because the inability to accurately identify when first doses of on-demand PrEP will be needed (e.g., variability in PrEP users’ circumstances surrounding the initiation of on-demand dosing) precludes making practical suggestions to support correct use | PrEP providers find it easy to support PrEP users to adhere to their chosen regimen because they can offer practical suggestions to help PrEP users remember to take daily PrEP and the ‘after’ doses, if using on-demand PrEP | “*With daily dosing it’s like with the pill, we tell them you can put an alarm on your phone or something or if you’re on another medicine, take it at the same time, you know, just ways that they can remember it. The event-based one is trickier, you know, to think of triggers that can help them remember to do it*.” (Sexual healthcare professional) | Skills  Environmental context and resources | Training  Education  Enablement | 4.4 Behavioural experiments  4.1 Instruction on how to perform the behaviour | 41. Sexual healthcare professionals could suggest that people using on-demand PrEP test different approaches to trigger their initial dose and note which approach is most successful (4.4)  41. Sexual health services should provide sexual healthcare professionals with a list of practical tips (e.g. in a national patient information booklet, wallet-sized insert) that they can share and discuss with PrEP users to encourage adherence to a daily PrEP regimen or the ‘after’ doses, if using on-demand PrEP (4.1) | 41. Accept – merge with others marked 41  41. Accept – add a new sentence at the end ‘Practical tips could include advising PrEP users to:’ and then list other recommendations marked 41 | (PA1i) PrEP services should provide PrEP providers and NGO staff with a list of practical tips for taking PrEP to share with PrEP users. *Strategies for daily PrEP and the ‘after’ doses of on-demand PrEP include: formulating an ‘if-then’ plan that links taking PrEP once a day to a specific task (e.g. brushing teeth) which remains constant even in the absence of or disruption to a daily routine; marking PrEP use on a calendar or recording it in a diary; setting reminder alarms and/or using a pill organiser; and keeping PrEP handy by carrying it and/or storing it in convenient places. A strategy for starting on-demand PrEP could be to test different approaches to trigger the initial dose and note which approach is the most successful* |
| PrEP providers find it difficult to support PrEP users to adhere to their chosen regimen because of the complexity of and unfamiliarity with on-demand dosing (e.g. when to start, stopping rules for different scenarios) | PrEP providers find it easy to support PrEP users to adhere to their chosen regimen because nationally-developed patient information booklets with key points about the various ways to take PrEP and diagrams showing how to follow on-demand PrEP aid provision of accurate dosing advice | “*I don’t know how good I would be if they were saying so I’m going to have sex on a Saturday and then I’m going to have sex on a Thursday, when do I actually start and stop it, you know. So, it’s case-by-case and I probably still need to refresh my memory a little bit and read up a bit on that still if I was doing that because most of the people are just taking it every day*.” (Sexual healthcare professional)  “*Having something like a patient information leaflet just allows you to codify your advice very clearly which is actually more useful to clinicians than we give credit for*.” (Sexual healthcare professional) | Knowledge  Memory, attention, and decision processes  Environmental context and resources | Education  Enablement | 4.1 Instruction on how to perform a behaviour  7.1 Prompts/cues  2.7 Feedback on behaviour | 22. Use a multi-method approach to educate sexual healthcare professionals about on-demand dosing (4.1) and assist them during consultations (7.1). For example, include clear written instructions and diagrams that depict how to take on-demand PrEP, including examples of when to start and stop for various scenarios, in a range of resources (e.g. brief fact sheet, PrEP provider pocket guide, national patient information booklets), provide sexual healthcare professionals with laminated copies of the on-demand dosing diagrams that they can pin to their wall as a quick reminder of how to use on-demand PrEP, record a short video or soundbite that explains on-demand dosing for different scenarios that sexual healthcare professionals may watch or listen to at a future date, include an online or paper-based quiz with questions about on-demand dosing as part of sexual healthcare professionals PrEP training and ongoing CPD and ensure that there is sufficient time or a named person to contact to discuss the answers after, if necessary (2.7) | 22. Modify – lots of nice examples to provide detail to headline recommendations. Consider who hosts the resources/ joined up approach/ ensure not developed piece-meal in different regions at same time. Need user involvement to tailor resources to key populations and ensure issues of relevance are covered | (PA1ii) PrEP services should use a joined-up, multi-method approach to improve PrEP providers’ understanding of on-demand dosing to assist them during consultations. *The following approaches could help: a range of resources (e.g. national, co-produced PrEP provider pocket guide and patient information, short videos, wall-mounted displays) with clear written instructions and visuals depicting correct usage of on-demand PrEP, including examples of when to start and stop for various scenarios, and a quiz with questions about on-demand dosing as part of PrEP training* |
