# Supplementary material for: Improving HIV pre-exposure prophylaxis (PrEP) adherence and retention in care: Process evaluation and recommendation development from a nationally implemented PrEP programme
Source: PLoS One. 2023 Oct 9;18(10):e0292289. doi: 10.1371/journal.pone.0292289 (PMC10561843; doi:10.1371/journal.pone.0292289)
Supplement: S2 Table — (DOCX) [file pone.0292289.s002.docx]

**S2 Table. Priority area 2 - A BCW analysis of ‘PrEP users consistently take PrEP appropriately’**

| **Barriers** | **Facilitators** | **Indicative quotes** | **TDF domains** | **Intervention Functions** | **Potential BCTs**  from the BCTTv1 (Michie et al. 2013) | **Initial recommendations for those considering implementing PrEP at scale**  Numbers in brackets = BCTs | **Post-APEASE and expert input decision**  Accept/Reject/Modify | **Agreed final recommendations** **for those considering implementing PrEP at scale** |
| --- | --- | --- | --- | --- | --- | --- | --- | --- |
| PrEP users find it difficult to consistently take PrEP appropriately because of the absence of or disruption to a daily or usual routine and inability to predict when sex will occur to trigger first dose for on-demand users | PrEP users find it easy to consistently take PrEP appropriately because they incorporate  taking PrEP into a pre-existing daily routine, if taking PrEP once a day (e.g. having breakfast, brushing teeth, taking other medication or vitamins if taking PrEP once a day) or a usual routine ahead of planned sex, if using on-demand PrEP | “*I do take medication already, daily, other mediation, so it's just an extra tablet in the morning. If it had been the more complicated dosage, of like, you know, two tables, and doing one and one, I would probably get more confused. But the one daily fits in pretty well with my lifestyle*.” (PrEP user) | Memory, attention and decision processes  Environmental context and resources  Behavioural regulation | Environmental restructuring  Enablement | 7.1 Prompts/cues  1.4 Action planning  2.3 Self-monitoring of behaviour | 41. Advise PrEP users to formulate an ‘if-then’ plan that links taking PrEP once a day to a specific task which remains constant even in the absence of or disruption to a daily routine (1.4, 7.1)  41. Suggest that PrEP users mark off on a calendar or record in a diary whether they have taken their daily medication (2.3)  Sexual healthcare professionals, other HCPs providing PrEP care, and NGO staff should share this practical tip with PrEP users. Practical tips should also appear in national patient information booklets and online resources (e.g. sexual health services, NGO, and HIV/PrEP activists’ websites and social media) | 41. Accept – merge with others marked 41  Rec merged with others marked 41 including those in priority area 1 ‘PrEP providers support PrEP users to adhere to their chosen regimen’. Did not include that final rec in this priority area after de-duplication (felt more appropriate in priority area 1 and to include the final rec to the right in this priority area instead)  41. Accept – merge with others marked 41  Rec merged with others marked 41 including those in priority area 1 ‘PrEP providers support PrEP users to adhere to their chosen regimen’. Did not include that final rec in this priority area after de-duplication (felt more appropriate in priority area 1 and to include the final rec to the right in this priority area instead) | (PA2v) PrEP users should consider a range of strategies, including those outlined in priority area one, to ensure effective use of PrEP and share those they find beneficial with potential and other PrEP users |
| PrEP users find it difficult to consistently take PrEP appropriately because inflexible clinic appointment processes owing to staff capacity mean PrEP users can run low on or out of PrEP | -- | “*The difficulty is where you have DNAs or people just choosing to come to the walk-in clinic for follow-up PrEP and the nursing team not being in a position to be able to do that and being able to manage patient expectations in terms of that consultation. And so, it’s about trying to reinforce with the patient that the follow-up is at these dedicated times, certainly with their agreement, but they have to come to those appointments for their further prescriptions to be given, you can’t just pop up on the off-chance that you’ll be given a further supply of PrEP. So, it’s been about that, managing the DNAs and then trying to fit them in somewhere else and already stretched clinics and them saying they’re running out of medication and then you feeling duty bound to try your best, to try and ensure they don’t have gaps in the provision of the medication*.” (Sexual healthcare professional) | Environmental context and resources | Environmental restructuring | 12.1 Restructure the physical environment | 00. Sexual healthcare professionals should support PrEP users to navigate services for appropriate expert support. Support could include providing clear information on how to get further PrEP prescriptions (i.e. clinic-specific processes, managing expectations - PrEP not an emergency, try and plan appointments in advance as clinics can fill up quickly)  2. Establish PrEP as routine clinical practice within sexual health services and implement PrEP reviews through regular drop-in clinics, in addition to booked appointments (12.1) | 00. Accept. Merged with 37 and 45 to create the final recommendation  2. Accept – flexible service for those whom fixed appointments do not suit. Will improve access to reviews but could be issues re: staff competencies and time, especially if there are things on “shopping list”, such as symptoms of STIs * Duplicate and merged with 4 and 6a which also relate to flexible provision of individualised PrEP care that meets diverse needs – included as a final rec for PA3 ‘PrEP users attend PrEP reviews’ (as 4 and 6a relate to this PA too) rather than for this PA | (PA2iv) PrEP providers and NGO staff (potentially through the use of peer navigators) should support PrEP users to navigate services and online information for appropriate expert support. *Support could include: providing clear information on how to get further PrEP prescriptions (i.e. clinic-specific processes, managing expectations - PrEP not an emergency, try and plan appointments in advance as clinics can fill up quickly); ensuring PrEP users know they can return to or call the PrEP service for adherence support and have the option to change regimens; and raising awareness of and directing PrEP users to reputable online sources of adherence support* |
| **--** | PrEP users find it easy to consistently take PrEP appropriately because they receive adherence support from sexual healthcare professionals (e.g. at PrEP review appointments, through provision of nationally-developed patient information booklets, on an ad-hoc basis) | “*The first question, it's looking at adherence, have they had any side-effects, or have they managed to take it, are they remembering it every day. Or if its event based, are they remembering to take it as they should. Is event based still the thing for them, do they want to change onto daily*.” (Sexual healthcare professional)  “*It was quite a visual leaflet…it would give almost like a timeline of how it would work, and it showed very clearly what the difference was between event based and also daily dosing, that was really, really helpful*. (PrEP user)  “*…keep the leaflet there so you can refer to it and what exactly you need to do and if you’ve got any problems, give us a phone. We always give out the details of the clinic for phoning for any queries*.” (Sexual healthcare professional) | Environmental context and resources  Professional role and identity  Social influences | Environmental restructuring  Education  Persuasion  Enablement | 7.1 Prompts/cues  5.1 Information about health consequences  9.1 Credible source  4.1 Instruction on how to perform the behaviour  1.1 Goal setting (behaviour)  1.4 Action planning  1.2 Problem solving  3.1 Social support (unspecified)  1.5 Review behavioural goal(s) | 16. Create paper-based or electronic checklists/proformas (based on formal protocols for PrEP initiation and review) that prompt sexual healthcare professionals to cover adherence-related issues at all PrEP appointments (7.1)  42. Sexual healthcare professionals must educate PrEP users on the importance of good adherence to ensure PrEP efficacy and minimise risks of HIV infection and antiretroviral resistance (5.1, 9.1)  43. Sexual healthcare professionals should provide PrEP users with verbal, written, and visual instructions re: medication dose, schedule, lead-in time to protection, and missed doses for the various ways of taking PrEP (e.g. via national patient information leaflet, wallet-sized insert) (4.1). Ensure these materials are also available in clinic waiting areas and at other relevant settings (e.g. NGOs)  44. At PrEP initiation, sexual healthcare professionals could set PrEP users a goal in terms of the behaviour to be achieved (e.g. daily dosing) (1.1, 1.4) and engage them in coping planning (1.2) to overcome barriers / increase facilitators to taking PrEP as per their chosen regimen. During PrEP reviews, sexual healthcare professionals should explore how well PrEP users have adhered to their chosen regimen (1.5) and with their agreement, either reset the same goal (i.e. stick to same regimen) or modify the future goal (i.e. switch regimen) (1.1)  45. Sexual healthcare professionals could direct PrEP users to reputable online sources of adherence support (e.g. sexual health services, NGO and HIV/ PrEP activists’ websites and social media) (3.1, 9.1) in addition to the information they provide (e.g. verbally, via provision of national patient information booklet)  37. Sexual healthcare professionals should inform PrEP users how to access the sexual health service for ad-hoc adherence support between appointments and ensure contact details and opening hours are kept up to date on the sexual health service website (3.1) | 16. Accept – could be an example of how to support adherence (relates to safe-care and people feeling that protocols are a good way of achieving this). Could be combined with other recommendations re: protocols  42. Accept – merge with other adherence-related recommendations  43. Accept – merge with other adherence-related recommendations  44. Modify – use professional judgement to decide whether an explicit exercise in goal setting and coping planning is required  45. Accept – flexible services that meet local population needs? Merged with 00 and 37 to create the final rec  37. Modify – don’t want to encourage people to have ad-hoc appointments but people do need the confidence to navigate the healthcare system (i.e. know they can come back to speak to a sexual healthcare professional, option to change regimens). Merged with 00 and 37 to create the final rec. Duplicate | (PA2i) PrEP services should create checklists/proformas, based on formal protocols, to prompt PrEP providers to cover adherence-related issues during PrEP initiation and reviews  (PA2ii) PrEP providers should emphasise the importance of adherence to minimise the risks of acquiring HIV and developing antiretroviral resistance and provide verbal, written, and visual instructions regarding medication dosing schedule, starting, stopping, and missed doses  (PA2iii) PrEP providers should consider offering PrEP users an explicit exercise in goal setting, coping planning (plans to deal with anticipated barriers to achieving these goals), and review of behavioural goals to support adherence to their chosen PrEP regimen  (PA2iv) PrEP providers and NGO staff (potentially through the use of peer navigators) should support PrEP users to navigate services and online information for appropriate expert support. *Support could include: providing clear information on how to get further PrEP prescriptions (i.e. clinic-specific processes, managing expectations - PrEP not an emergency, try and plan appointments in advance as clinics can fill up quickly); ensuring PrEP users know they can return to or call the PrEP service for adherence support and have the option to change regimens; and raising awareness of and directing PrEP users to reputable online sources of adherence support* |
| **--** | PrEP users find it easy to consistently take PrEP appropriately because they put in place reminders to avoid missing a dose (e.g. phone alarm or alert on an app, use of a pill organiser) | “*When your phone buzzes at 12 o'clock then you know it's time to take your pill. I've found that helps*.” (PrEP user)  *“I decided to use a dosette box and have all of my medications there and that’s kind of keeping me in check of taking them daily.”* (PrEP user) | Memory, attention and decision processes  Environmental context and resources  Behavioural regulation | Environmental restructuring  Enablement | 7.1 Prompts/cues  12.5 Adding objects to the environment | 41. Encourage PrEP users (e.g. during interactions with sexual healthcare professionals and NGO staff, via sexual health services, NGO, and HIV/PrEP activists’ websites and social media, in national patient information booklets) to set up reminder alarms (7.1) and/or use a pill organiser (7.1, 12.5) as prompts to take PrEP | 41. Accept – merge with others marked 41  Rec merged with others marked 41 including those in priority area 1 ‘PrEP providers support PrEP users to adhere to their chosen regimen’. Did not include that final rec in this priority area after de-duplication (felt more appropriate in priority area 1 and to include the final rec to the right in this priority area instead) | (PA2v) PrEP users should consider a range of strategies, including those outlined in priority area one, to ensure effective use of PrEP and share those they find beneficial with potential and other PrEP users |
| **--** | PrEP users find it easy to consistently take PrEP appropriately because they keep PrEP handy by carrying it on them (e.g. in a bag, jacket pocket) and storing it in convenient places around and outside the home (e.g. in the car, at work) | *“I keep it somewhere where I look through, like in my backpack, where I would look for many things…then if I forget, I will very soon see it, as in, see the box with the PrEP and be like, oh did I take it, oh yeah I did, or, oh no I didn’t.”* (PrEP user)  “*I would actually have one bottle in my bag, I’d have one in the living room and one in the bedroom so wherever I was during the day I could actually take it...because sometimes I’d remember and I’d think, oh, wait a minute, and if I was at work I could take it if I needed to. Because I’d have one in my bag*.” (PrEP user) | Memory, attention and decision processes  Environmental context and resources  Behavioural regulation | Environmental restructuring  Enablement | 12.1 Restructure the physical environment  7.1 Prompts/cues | 41. Advise PrEP users to keep PrEP handy by carrying it on them and storing it in convenient places around and outside the home (12.1), both as a prompt to take PrEP (7.1) and to ensure it is readily accessible  Sexual healthcare professionals, other HCPs providing PrEP care, and NGO staff should share this practical tip with PrEP users. Practical tips should also appear in national patient information booklets and online resources (e.g. sexual health services, NGO, and HIV/PrEP activists’ websites and social media) | 41. Accept – merge with others marked 41  Rec merged with others marked 41 including those in priority area 1 ‘PrEP providers support PrEP users to adhere to their chosen regimen’. Did not include that final rec in this priority area after de-duplication (felt more appropriate in priority area 1 and to include the final rec to the right in this priority area instead) | (PA2v) PrEP users should consider a range of strategies, including those outlined in priority area one, to ensure effective use of PrEP and share those they find beneficial with potential and other PrEP users |
