# Supplementary material for: Improving HIV pre-exposure prophylaxis (PrEP) adherence and retention in care: Process evaluation and recommendation development from a nationally implemented PrEP programme
Source: PLoS One. 2023 Oct 9;18(10):e0292289. doi: 10.1371/journal.pone.0292289 (PMC10561843; doi:10.1371/journal.pone.0292289)
Supplement: S3 Table — (DOCX) [file pone.0292289.s003.docx]

**S3 Table. Priority area 3 - A BCW analysis of ‘PrEP users attend PrEP reviews’**

| **Barriers** | **Facilitators** | **Indicative quotes** | **TDF domains** | **Intervention Functions** | **Potential BCTs**  from the BCTTv1 (Michie et al. 2013) | **Initial recommendations for those considering implementing PrEP at scale**  Numbers in brackets = BCTs | **Post-APEASE and expert input decision**  Accept/Reject/Modify | **Agreed final recommendations** **for those considering implementing PrEP at scale** |
| --- | --- | --- | --- | --- | --- | --- | --- | --- |
| PrEP users find it difficult to attend PrEP reviews because there are limited options for where (e.g. at some not all sexual health services, located far away), when (e.g. inconvenient time slots), and how (e.g. by appointment, set up to be delivered in male only or GBMSM clinics) they can access them | PrEP users find it easy to attend PrEP reviews because there is flexibility in where (e.g. at all sexual health services, in other more valued / acceptable settings), when (e.g. extended opening hours), and how (e.g. via drop-in clinics, by appointment) they can access them | *“They can't take the kidney tests in the [outreach] clinic that’s dedicated to gay men, because it's in a different venue…so, essentially, if at those clinics, if they could take the kidney test as well.”* (PrEP user)  “*Because we’re a rural area, we might have patients that live a distance away, and with work commitments, it might make it difficult for them to come into the clinic. Whereas if they live a distance away, but they happen to be in [town], then on a particular day, if they were able to just get it from a drop-in clinic, then it would be easier for them.*” (Sexual healthcare professional) | Environmental context and resources | Environmental restructuring  Enablement | 12.1 Restructure the physical environment  12.2 Restructure the social environment  3.1 Social support (unspecified) | 1. Consider alternative service models to make PrEP reviews available to PrEP users via a range of settings, including all sexual health services (e.g. local hubs and satellites, as well as central services), remote care (e.g. ePrEP, phone consultations), community venues (e.g. outreach clinics), and non-sexual health-specific health services (e.g. reproductive health clinics, GP surgeries), with agreed pathways for non-complex PrEP users and those with additional medical complexity (12.1, 12.2)  2. Establish PrEP as routine clinical practice within sexual health services and implement PrEP reviews through regular drop-in clinics, in addition to booked appointments (12.1)  3. Maximise all drop-in visits by ensuring there is sufficient waiting space, toilets, and consultation rooms (12.1) and operationalising drop-in clinics via a multidisciplinary team of sexual healthcare professionals who can task-share and accommodate complex cases (12.2)  4. Provide access to drop-in clinics and pre-bookable appointments on mid-week evenings and at weekends to suit contemporary lifestyles and meet local population needs (12.1)  5. Support PrEP users in becoming aware of when and how they can access drop-in clinics and book and reschedule appointments for PrEP reviews (e.g. sexual healthcare professionals provide information verbally, hand out location-specific leaflets or wallet-sized inserts, signpost to websites) (3.1) | 1. Modify – there has to be reasonable provision of PrEP throughout the wider healthcare system to extend reach, as otherwise some people will not benefit. Increased reach of PrEP could be viewed as aspirational. Too much detail at the moment, lose from ‘including all sexual health services’ to ‘GP surgeries’. Some concerns are that HCPs in non-specialist settings may not see many PrEP users, effectiveness, cost and monitoring, equity in terms of the digital divide *  2. Accept – flexible service for those whom fixed appointments do not suit. Will improve access to reviews but could be issues re: staff competencies and time, especially if there are things on “shopping list”, such as symptoms of STIs. * Duplicate. Merged with 4 and 6a which also relate to flexible provision of individualised PrEP care that meets diverse needs  3. Reject – what services should be doing anyway and an underlying assumption about what is available in order to support delivery of good SRH in general. Not specific to PrEP  4. Accept – relates to flexible service provision. * Merged with 2 and 6a  5. Reject – too general and already part of existing service | (PA3i) PrEP service planners should consider offering reviews in a range of settings (not solely sexual health clinics). *Each service model should incorporate pathways for non-complex PrEP users and those with additional medical complexity*  (PA3ii) PrEP services should ensure individualised PrEP care is provided flexibly to meet diverse needs. *Examples include: implementing PrEP reviews through drop-in clinics as well as booked appointments (as the programme matures); providing evening and weekend access to suit lifestyles and meet local population needs; ensuring there are options for how to book in for the next review (e.g. online, by phone, in-person), with the appointment system open far enough in advance to enable booking in before leaving the premises; and flexibility to provide extra PrEP supply to accommodate longer periods between reviews, if necessary* |
| PrEP users find it difficult to attend PrEP reviews because of an absence of appointment scheduling, reminder, follow-up, and/or other targeted intervention processes | PrEP users find it easy to attend PrEP reviews because there are appointment scheduling, reminder, follow-up, and/or other targeted intervention processes in place | *“Once you’re finished and got the PrEP you actually just make the appointment for the next three months at that point, and then you go back and you go through the tests again…”* (PrEP user)  “*They send a text the following day after the appointment’s made, and then they send a text two days prior to the appointment to confirm your time. So that works really well*.” (PrEP user)  “*If they have DNA’d the appointment we may give them a follow-up phone call, but if they have just not made another appointment then we don't follow them up*.” (Sexual healthcare professional) | Environmental context and resources  Memory, attention and decision processes  Behavioural regulation | Environmental restructuring  Enablement | 12.1 Restructure the physical environment  7.1 Prompts/cues  2.2 Feedback on behaviour  3.1 Social support (unspecified) | 6a. Ensure the appointment system is open far enough in advance to enable PrEP users to book their next PrEP appointment before leaving the premises (12.1)  7. Prompt sexual healthcare professionals (e.g. via paper-based or electronic checklists/ proformas, SOPs, ‘pop-up’ messages within the IT system) to remind PrEP users to book their next appointment before leaving the premises (7.1)  8. Use an automated reminder system to alert PrEP users (e.g. via email, voice message, SMS) to a booked appointment (7.1) or to notify that they are overdue to attend (2.2)  9. Integrate ‘pop-up’ messages into the IT system to inform sexual healthcare professionals that a PrEP user did not attend or are overdue a PrEP appointment and advise exploration of the issue (7.1)  10a. Run a monthly report on the IT system to identify ‘did not attends’ and those overdue a PrEP appointment and attempt to make contact with PrEP users (e.g. via email, SMS, phone) and reengage them with PrEP care, if appropriate (3.1) | 6a. Modify – Doesn’t need to be done physically at the time, could be done remotely. Key point is to ensure availability of next slot at required time and ability to “flex” the timing if necessary. For example, extra PrEP supply if away somewhere or unsure. May not suit those who have less fixed plans. Does make it easier to follow up defaulters. Merged with 2 and 4  7. Reject – kill all pop-ups. Already happens. Duplicate  8. Modify – may not be acceptable for everyone so need to have opt-out. Need reminder in advance of an upcoming review and further prompt if overdue  9. Reject – favour automated system which sends reminders to PrEP users. Duplicate  10a. Accept – about knowing your PrEP cohort (at service-level). Findings can inform PrEP initiation process (develop interventions for those who miss appointments/ are more likely to not reattend to improve retention rates) | (PA3ii) PrEP services should ensure individualised PrEP care is provided flexibly to meet diverse needs. *Examples include: implementing PrEP reviews through drop-in clinics as well as booked appointments (as the programme matures); providing evening and weekend access to suit lifestyles and meet local population needs; ensuring there are options for how to book in for the next review (e.g. online, by phone, in-person), with the appointment system open far enough in advance to enable booking in before leaving the premises; and flexibility to provide extra PrEP supply to accommodate longer periods between reviews, if necessary*  (PA3iii) PrEP services should use existing or introduce new clinic processes, such as an automated text message system (with opt-out option), to remind and follow-up PrEP users about PrEP reviews and to try and reengage non-attenders.  (PA3iv) PrEP services should consider their patient cohort alongside the available evidence to identify characteristics of people likely to miss appointments or not re-attend for PrEP reviews and develop interventions to be delivered at PrEP initiation to improve retention in care |
| PrEP users find it difficult to attend PrEP reviews because they do not require a PrEP prescription (e.g. they are doing on-demand dosing or have stopped PrEP in the interim period) | PrEP users find it easy to attend PrEP reviews because they value the regular sexual health screening and other health tests and discussions that take place within PrEP reviews | “*It's sometimes a struggle to get them back at three months for review. What brings them back essentially is wanting more medication and actually if they don't want more medication they're less inclined to return for review, and obviously if they've had any risky sexual behaviour then they should be getting an STI screen. So, it can be difficult to get those men back*.” (Sexual healthcare professional)  “*If you’re constantly getting kidney and liver function tests and it comes back positive, then everything’s working fine…so, that kind of reassures me about my health. I think they’re very important*.” (PrEP user) | Beliefs about consequences  Behavioural regulation | Education  Persuasion  Enablement | 5.1 Information about health consequences  5.6 Information about emotional consequences  9.1 Credible source  1.8 Behavioural contract  1.9 Commitment  1.1 Goal setting (behaviour) | 31a. Provide PrEP users with a range of information sources (e.g. posters, national patient information booklets, positive testimonials of PrEP users, online resources, verbal communication by sexual healthcare professionals and NGO staff) regarding the health and emotional benefits of PrEP reviews, including the importance of regular HIV and STI testing and discussing stopping PrEP with a sexual healthcare professional (5.1, 5.6, 9.1)  01. Sexual healthcare professionals could ask PrEP users to verbally agree to or sign a written contract specifying that they will attend for regular PrEP reviews, even if they are doing on-demand dosing or have stopped PrEP in the interim period (1.8, 1.9, 1.1) | 31a. Modify –easy/inexpensive though not PrEP specific, would apply to any chronic condition, and will have a small diminishing effect. Ideally info should be co-produced and tailored for different populations. Also include the importance of renal monitoring and review of how things are going. One resource for use across PrEP continuum  01. Modify – impractical and too paternalistic in current form. Duplicate | (PA3v) PrEP providers and NGO staff should encourage optimal PrEP use by emphasising the health and emotional benefits of PrEP reviews, such as regular HIV and STI testing, renal monitoring and review of ‘how things are going’, and the importance of discussing stopping PrEP with a PrEP provider. *Information sources may include co-produced patient information and verbal communication*  (PA3vi) PrEP users should commit to engaging with regular PrEP reviews, even if they do not require a new PrEP prescription when the next review is due |
| -- | PrEP users find it easy to attend PrEP reviews because PrEP providers are explicit about the requirement for PrEP reviews at the outset | “*They kind of make this agreement. So, if you want to be on PrEP funded by the NHS, this is the expectation …you come every three months and we do this, this and this and if you don’t come every three months, if you miss your appointments, you may fall off the protocol to still be funded for PrEP. So, it’s very much like an agreement. So, all that is definitely explained and set out to them at the initial assessment*.” (Sexual healthcare professional) | Reinforcement  Behavioural regulation | Coercion  Enablement | 10.11 Future punishment  1.8 Behavioural contract  1.9 Commitment  1.1 Goal setting (behaviour) | 32. Sexual healthcare professionals should inform PrEP users at the initial assessment that they risk losing their access to PrEP if they do not attend for regular PrEP reviews (10.11)  01. Sexual healthcare professionals could ask PrEP users to verbally agree to or sign a written contract specifying that they will attend for regular PrEP reviews, even if they are doing on-demand dosing or have stopped PrEP in the interim period (1.8, 1.9, 1.1) | 32. Reject – too paternalistic and not person-centred. Feels punitive and controlling  01. Modify – impractical and too paternalistic in current form  Duplicate | (PA3vi) PrEP users should commit to engaging with regular PrEP reviews, even if they do not require a new PrEP prescription when the next review is due |
