# Supplementary material for: Improving HIV pre-exposure prophylaxis (PrEP) adherence and retention in care: Process evaluation and recommendation development from a nationally implemented PrEP programme
Source: PLoS One. 2023 Oct 9;18(10):e0292289. doi: 10.1371/journal.pone.0292289 (PMC10561843; doi:10.1371/journal.pone.0292289)
Supplement: S4 Table — (DOCX) [file pone.0292289.s004.docx]

**S4 Table. Priority area 4 - A BCW analysis of ‘PrEP providers reassess PrEP users’ candidacy based on risk of HIV acquisition’**

| **Barriers** | **Facilitators** | **Indicative quotes** | **TDF domains** | **Intervention Functions** | **Potential BCTs**  from the BCTTv1 (Michie et al. 2013) | **Initial recommendations for those considering implementing PrEP at scale**  Numbers in brackets = BCTs | **Post-APEASE and expert input decision**  Accept/Reject/Modify | **Agreed final recommendations** **for those considering implementing PrEP at scale** |
| --- | --- | --- | --- | --- | --- | --- | --- | --- |
| PrEP providers find it difficult to reassess PrEP users’ candidacy based on risk of HIV acquisition because they overlook this aspect of PrEP reviews (e.g. familiarity and routinisation of giving out PrEP, assume PrEP users have an ongoing need) | PrEP providers find it easy to reassess PrEP users’ candidacy based on risk of HIV acquisition because supporting documents and the IT system prompt them to undertake this task | “*The danger to that is, because* *you can get a bit complacent about it and think that this is just doing tests and handing out drugs, and not properly reviewing people… checking that they still fit the eligibility criteria, and things like that*.” (Sexual healthcare professional)  *“They didn’t really go into depth to see if I was still eligible. They kind of assumed.”* (PrEP user)  “*She created template documents for us to use that would prompt us to ask the right questions, consider the right things, and we had one for initial assessment, we had one for a one-month review and we had one for a three-month review*.” (Sexual healthcare professional) | Memory, attention and decision processes  Environmental context and resources  Behavioural regulation | Environmental restructuring  Enablement  Persuasion | 7.1 Prompts/cues  2.3 Self-monitoring of behaviour  2.4 Self-monitoring of outcome(s) of behaviour  5.1 Information about health consequences  9.1 Credible source | 11. Create paper-based or electronic checklists/ proformas (based on a formal protocol for PrEP reviews) that prompt sexual healthcare professionals to reassess PrEP users’ eligibility (7.1) and request documentation of their eligibility decision (2.3) with a record of the reasons to support it (2.4)  12. Introduce interactive ‘pop-up’ messages within the IT system (7.1) that alert sexual healthcare professionals to the potential health risks of PrEP (5.1) and require them to confirm they have reassessed PrEP users’ eligibility (2.3) prior to prescribing  17. Use a multi-method approach to educate sexual healthcare professionals about the importance of reassessing PrEP users’ eligibility to avoid them taking PrEP unnecessarily if they are no longer at high risk for HIV acquisition (5.1, 9.1) | 11. Reject– could help those who may wish to consider stopping PrEP or changing regimen. But tick boxes often dehumanise and impair proper engagement and discussion plus more concern that PrEP would be removed from people still at risk since current eligibility criteria / risk assessments aren’t sensitive enough for all key populations  12. Reject – staff hate pop-ups  17. Reject – basic competence. Also, as PrEP users move between initiation (face to face) to follow-up (remote?), its perhaps better to have annual check ins where these issues can be addressed | -- |
