# Supplementary material for: Improving HIV pre-exposure prophylaxis (PrEP) adherence and retention in care: Process evaluation and recommendation development from a nationally implemented PrEP programme
Source: PLoS One. 2023 Oct 9;18(10):e0292289. doi: 10.1371/journal.pone.0292289 (PMC10561843; doi:10.1371/journal.pone.0292289)
Supplement: S5 Table — (DOCX) [file pone.0292289.s005.docx]

**S5 Table. Priority area 5 - A BCW analysis of ‘PrEP providers address wider sexual health issues’**

| **Barriers** | **Facilitators** | **Indicative quotes** | **TDF domains** | **Intervention Functions** | **Potential BCTs**  from the BCTTv1 (Michie et al. 2013) | **Initial recommendations for those considering implementing PrEP at scale**  Numbers in brackets = BCTs | **Post-APEASE and expert input decision**  Accept/Reject/Modify | **Agreed final recommendations** **for those considering implementing PrEP at scale** |
| --- | --- | --- | --- | --- | --- | --- | --- | --- |
| PrEP providers find it difficult to address wider sexual health issues because of the time constraints of PrEP review appointments | PrEP providers find it easy to address wider sexual health issues because they have generous and/or flexible appointment times for PrEP reviews | *“These can potentially be quite lengthy and complex dialogues that aren't necessarily going to be able to be accommodated within a short consultation on a three-monthly basis.”* (NGO staff)  “*In [urban Health Board], I know they're really pushed for time in the PrEP clinics, whereas here, we are a bit more flexible, and we can kind of, we have time to chat as much or as little as they want*.” (Sexual healthcare professional) | Environmental context and resources  Professional role and identity | Environmental restructuring  Education  Persuasion  Enablement | 12.2 Restructure the social environment  5.1 Information about health consequences  5.3 Information about social and environmental consequences  5.6 Information about emotional consequences  2.3 Self-monitoring of behaviour  2.4 Self-monitoring of outcome(s) of behaviour  2.2 Feedback on behaviour  2.7 Feedback on outcome(s) of behaviour  3.1 Social support (unspecified) | 13. Sexual health services should explore and provide innovative ways of scheduling appointments with built-in flexibility to respond to long standing health inequalities in health and HIV literacy and varying needs of PrEP users (e.g. longer discussions about PrEP and wider sexual health issues) (12.2)  23. Facilitate and sustain an organisational culture that values a wholistic approach to sexual health and wellbeing (12.2) (e.g. reflect a wholistic approach in the sexual health service values and mission statement and include as a core competency for professional conduct, address in education sessions (5.1, 5.3, 5.6) and reflective practice, and as part of annual appraisals (2.3, 2.4, 2.2, 2.7))  27. Check that PrEP users are aware of other specialist services available locally (e.g. delivered by NGOs, available within the sexual health service) and signpost or make a direct referral, as necessary (3.1) | 13. Modify – not PrEP specific but providing individualised PrEP care and responding to long standing health inequalities in health and HIV literacy and varying need is important and needs to feature somewhere (flexibility of service provision). ‘Innovative’ is too subjective. Duplicate  23. Reject – don’t dispute its importance, but favour other PrEP-specific recommendations. This should already be embedded in good clinical practice. Duplicate  27. Modify – reciprocity of connection that is in stage 1. Duplicate | (PA5i) PrEP services should ensure flexible provision of individualised PrEP care that meets diverse needs. *For example, explore and provide ways of scheduling appointments with built-in flexibility to respond to long-standing inequalities in health and HIV/PrEP literacy during consultations*  (PA5iii) PrEP providers and NGO staff (potentially through the use of peer navigators) should support PrEP users to navigate services and online information for appropriate expert support. *Support could include signposting and/or referring PrEP users to other specialist services across and beyond the HIV prevention and care sector, as necessary* |
| -- | PrEP providers find it easy to address wider sexual health issues because they have built trusting relationships and familiarity with PrEP users through continuity of care | “*I certainly feel the…you know, the advantage to it, because you know them and they feel comfortable to tell you things and you feel comfortable to ask them things and you, kind of, know what’s been going on. You know, because, you saw them last time, so you know the questions that you asked ...and you, kind of, pick up where you left off*.” (Sexual healthcare professional) | Environmental context and resources  Social influences  Professional role and identity | Environmental restructuring  Education  Modelling  Enablement | 12.2 Restructure the social environment  12.1 Restructure the physical environment  7.1 Prompts/cues  5.1 Information about health consequences  5.3 Information about social and environmental consequences  6.1 Demonstration of the behaviour  2.2 Feedback on behaviour  2.3 Self-monitoring of behaviour | 14. Where possible, assign each PrEP user a ‘usual' sexual healthcare professional and operate a buddy system where paired sexual healthcare professionals can see each other's patients, for example, when the other is on leave, to facilitate continuity of care (12.2)  6b. Ensure the appointment system is open and the rota agreed far enough in advance to enable PrEP users to book their next PrEP appointment with their ‘usual’ sexual healthcare professional or buddy before leaving the premises (12.1)  7. Prompt sexual healthcare professionals (e.g. via paper-based or electronic checklists/ proformas, SOPs, ‘pop-up’ messages within the IT system) to remind PrEP users to book their next appointment before leaving the premises (7.1)  24. Facilitate and actively maintain (e.g. via training, huddles, clinical supervision, reflective practice) a warm, welcoming, and friendly atmosphere wherein sexual healthcare professionals communicate with patients in a non-judgemental manner, using inclusive, sex- and PrEP-positive, and destigmatising language to establish trust and ensure an open dialogue (12.2, 5.3)  18. Promote the advantages of high-quality clinical record keeping for continuity of care (5.1, 5.3), share best practice examples that meet the standards set out by the sexual health service and/or relevant professional bodies (6.1), and appraise and encourage sexual healthcare professionals to reflect on their skills of recording episodes of care (2.2, 2.3) | 14. Reject – totally impractical in the real world. Also, would limit training opportunities and foster dependence. Duplicate  6b. Reject – impractical for PrEP users to have a designated sexual healthcare professional for reviews. The bit about the appointment system being open in advance is covered in 6a. Duplicate  7. Reject – kill all pop-ups. Already happens. Duplicate  24. Reject – support the general sentiment but is not PrEP specific. Useful content for the intro as we will need to make a statement pointing towards existing standards / expectations of the bedrock of delivery. Duplicate  18. Reject – not PrEP specific. Is addressed in existing clinical governance, appraisal and revalidation. Duplicate | -- |
| -- | PrEP providers find it easy to address wider sexual health issues because they are trained to deliver brief behaviour change interventions or have the option to signpost PrEP users and/or make direct referrals to other specialist services (e.g. for drug and alcohol problems, gender clinic, rape/sexual assault) for appropriate support | “*It definitely feels better than it did years and years ago when I started. It was a bit like I don’t even know what to say now. I don’t even know. You know, you told me this awful thing and I feel I want to have an answer for you and I don’t know what the answer is. So, I do feel like there’s more accessible support that you could refer someone to*.” (Sexual healthcare professional) | Skills  Professional role and identity  Environmental context and resources | Training  Modelling  Environmental restructuring  Enablement | 4.1 Instruction on how to perform the behaviour  6.1 Demonstration of the behaviour  8.1 Behavioural practice/rehearsal  2.2 Feedback on behaviour  2.3 Self-monitoring of behaviour  12.2 Restructure the social environment  3.1 Social support (unspecified) | 19. Develop sexual healthcare professionals’ skills in delivering brief behaviour change interventions through interactive activities (e.g. via workshops, online courses, in clinical supervision), including training on the technical aspects (4.1), video examples and shadowing of more experienced sexual healthcare professionals for ‘what works’ tips (6.1), role-/real-play exercises (8.1) with provision of feedback (2.2), and ongoing reflections on skill acquisition (2.3)  25. Establish good connections with other specialist services (e.g. delivered by NGOs, those available within the sexual health service) (12.2) that sexual healthcare professionals could signpost and/or directly refer PrEP users to, for appropriate expert support (3.1)  26. Develop and raise sexual healthcare professionals’ awareness of protocols to ensure standardisation in care navigation (e.g. signposting and referrals to other specialist services) (4.1) | 19. Reject – not PrEP specific. Support in general but for broader delivery of quality care  25. Modify – Two-way connections / partnership work but need to word carefully so doesn’t seem like sexual health services are passing the buck and being mindful of 3^rd^ sector funding cuts. Include NGOs that serve communities other than GBMSM. Duplicate  26. Reject | (PA5ii) PrEP services and NGOs should enhance and maintain good connections across HIV prevention and care and other specialist services, to facilitate easy reciprocal referrals. *Consider carefully the type of support required and which service is best placed to provide it* |
