# Supplementary material for: Improving HIV pre-exposure prophylaxis (PrEP) adherence and retention in care: Process evaluation and recommendation development from a nationally implemented PrEP programme
Source: PLoS One. 2023 Oct 9;18(10):e0292289. doi: 10.1371/journal.pone.0292289 (PMC10561843; doi:10.1371/journal.pone.0292289)
Supplement: S6 Table — (DOCX) [file pone.0292289.s006.docx]

**S6 Table. Priority area 6 - A BCW analysis of ‘PrEP users discuss wider sexual health issues’**

| **Barriers** | **Facilitators** | **Indicative quotes** | **TDF domains** | **Intervention Functions** | **Potential BCTs**  from the BCTTv1 (Michie et al. 2013) | **Initial recommendations for those considering implementing PrEP at scale**  Numbers in brackets = BCTs | **Post-APEASE and expert input decision**  Accept/Reject/Modify | **Agreed final recommendations** **for those considering implementing PrEP at scale** |
| --- | --- | --- | --- | --- | --- | --- | --- | --- |
| PrEP users find it difficult to discuss wider sexual health issues because PrEP reviews feel rushed and are typically focused on PrEP only | -- | “*They don’t really say, well, you know, what’s your…what are you currently up to? Are you seeing anyone or…you know, there’s no, kind of, counselling service, if that makes… if that’s the right term to use. There’s no, kind of, how are you in your life and how are you within your sexual health, kind of thing. There’s none of that at all*.” (PrEP user) | Environmental context and resources  Professional role and identity | Environmental restructuring  Education  Persuasion  Enablement | 12.2 Restructure the social environment  5.1 Information about health consequences  5.3 Information about social and environmental consequences  5.6 Information about emotional consequences  2.3 Self-monitoring of behaviour  2.4 Self-monitoring of outcome(s) of behaviour  2.2 Feedback on behaviour  2.7 Feedback on outcome(s) of behaviour  3.1 Social support (unspecified) | 13. Sexual health services should explore and provide innovative ways of scheduling appointments with built-in flexibility to respond to long standing health inequalities in health and HIV literacy and varying needs of PrEP users (e.g. longer discussions about PrEP and wider sexual health issues) (12.2)  23. Facilitate and sustain an organisational culture that values a wholistic approach to sexual health and wellbeing (12.2) (e.g. reflect a wholistic approach in the sexual health service values and mission statement and include as a core competency for professional conduct, address in education sessions (5.1, 5.3, 5.6) and clinical supervision, and as part of annual appraisals (2.3, 2.4, 2.2, 2.7))  25. Establish good connections with other specialist services (e.g. delivered by NGOs, those available within the sexual health service) (12.2) that sexual healthcare professionals could signpost and/or directly refer PrEP users to, for appropriate expert support (3.1)  27. Check that PrEP users are aware of other specialist services available locally (e.g. delivered by NGOs, available within the sexual health services) and signpost or make a direct referral, as necessary (3.1) | 13. Modify – not PrEP specific but providing individualised PrEP care and responding to long standing health inequalities in health and HIV literacy and varying need is important and needs to feature somewhere (flexibility of service provision). ‘Innovative’ is too subjective. Duplicate  23. Reject. Duplicate  25. Modify – Two-way connections / partnership work but need to word carefully so doesn’t seem like sexual health services are passing the buck and being mindful of 3^rd^ sector funding cuts. Include NGOs that serve communities other than GBMSM. Duplicate  27. Modify – reciprocity of connection that is in stage 1. Duplicate | (PA6i) PrEP services should ensure flexible provision of individualised PrEP care that meets diverse needs. For example, explore and provide ways of scheduling appointments with built-in flexibility to respond to long-standing inequalities in health and HIV/PrEP literacy during consultations  (PA6ii) PrEP services and NGOs should enhance and maintain good connections across HIV prevention and care and other specialist services, to facilitate easy reciprocal referrals. *Consider carefully the type of support required and which service is best placed to provide it*  (PA6iii) PrEP providers and NGO staff (potentially through the use of peer navigators) should support PrEP users to navigate services and online information for appropriate expert support. *Support could include signposting and/or referring PrEP users to other specialist services across and beyond the HIV prevention and care sector, as necessary* |
| -- | PrEP users find it easy to discuss wider sexual health issues because they have built a trusting relationship and familiarity with PrEP providers through continuity of care | “*R: If I go and see him, at the [clinic], he knows my situation, he's actually really good on just being able to advise. If I go to the [clinic], it's completely luck of the draw who I get. So sometimes they'll have seen me five, six years ago, and won't remember me at all. It's better having the continuity, I think.*  *I: In terms of why it's better, what does it change for you?*  *R: It just feels safer, actually, there's a bond, there's a trust going on there… I mean, you should be able to trust a doctor, but for some reason, I find actually speaking to someone that I've known for a while, actually, I feel a lot more comfortable about that*.” (PrEP user) | Environmental context and resources  Social influences  Professional role and identity | Environmental restructuring  Training  Education  Modelling  Enablement | 12.2 Restructure the social environment  12.1 Restructure the physical environment  7.1 Prompts/cues  5.1 Information about health consequences  5.3 Information about social and environmental consequences  6.1 Demonstration of the behaviour  2.2 Feedback on behaviour  2.3 Self-monitoring of behaviour | 14. Where possible, assign each PrEP user a ‘usual' sexual healthcare professional and operate a buddy system where paired sexual healthcare professionals can see each other's patients, for example, when the other is on leave, to facilitate continuity of care (12.2)  6b. Ensure the appointment system is open and the rota agreed far enough in advance to enable PrEP users to book their next PrEP appointment with their ‘usual’ sexual healthcare professional or buddy before leaving the premises (12.1)  7. Prompt sexual healthcare professionals (e.g. via paper-based or electronic checklists/ proformas, SOPs, ‘pop-up’ messages within the IT system) to remind PrEP users to book their next appointment before leaving the premises (7.1)  24. Facilitate and actively maintain (e.g. via training, huddles, clinical supervision) a warm, welcoming, and friendly atmosphere wherein sexual healthcare professionals communicate with patients in a non-judgemental manner, using inclusive, sex- and PrEP-positive, and destigmatising language to establish trust and ensure an open dialogue (12.2, 5.3)  18. Promote the advantages of high-quality clinical record keeping for continuity of care (5.1, 5.3), share best practice examples that meet the standards set out by the sexual health service and/or relevant professional bodies (6.1), and appraise and encourage sexual healthcare professionals to reflect on their skills of recording episodes of care (2.2, 2.3) | 14. Reject. Duplicate  6b. Reject – impractical for PrEP users to have a designated sexual healthcare professional for reviews. The bit about the appointment system being open in advance is covered in 6a. Duplicate  7. Reject – kill all pop-ups. Already happens. Duplicate  24. Reject– support the general sentiment but is not PrEP specific. Useful content for the intro as we will need to make a statement pointing towards existing standards / expectations of the bedrock of delivery Duplicate  18. Reject – not PrEP specific. Is addressed in existing clinical governance, appraisal and revalidation. Duplicate | -- |
