# Supplementary material for: Improving HIV pre-exposure prophylaxis (PrEP) adherence and retention in care: Process evaluation and recommendation development from a nationally implemented PrEP programme
Source: PLoS One. 2023 Oct 9;18(10):e0292289. doi: 10.1371/journal.pone.0292289 (PMC10561843; doi:10.1371/journal.pone.0292289)
Supplement: S7 Table — (DOCX) [file pone.0292289.s007.docx]

**S7 Table. Priority area 7 - A BCW analysis of ‘PrEP users stay on PrEP for as long as relevant’**

| **Barriers** | **Facilitators** | **Indicative quotes** | **TDF domains** | **Intervention Functions** | **Potential BCTs**  from the BCTTv1 (Michie et al. 2013) | **Initial recommendations for those considering implementing PrEP at scale**  Numbers in brackets = BCTs | **Post-APEASE and expert input decision**  Accept/Reject/Modify | **Agreed final recommendations** **for those considering implementing PrEP at scale** |
| --- | --- | --- | --- | --- | --- | --- | --- | --- |
| PrEP users find it difficult to stay on PrEP for as long as relevant because they experience or are concerned about side-effects (e.g. allergy, rash, dry mouth, GI upset, spots, longer-term renal toxicity) | -- | “*I expected those kinds of symptoms with dry mouth and the wee bit funny queasiness maybe but in reality, it was a lot more intense and a lot worse than what I anticipated*.” (Stopped using PrEP) | Beliefs about consequences  Behavioural regulation | Education  Enablement | 5.1 Information about health consequences  1.2 Problem solving  2.6 Biofeedback  9.2 Pros and cons  3.1 Social support | 35. Educate PrEP users (e.g. verbally at PrEP appointments, in national patient information booklets, via sexual health services, NGO, and HIV/PrEP activists’ websites and social media) about the potential side-effects of PrEP and their typically transient nature (5.1), share management strategies for the most common side-effects (1.2), and reassure against concerns about longer-term toxic effects by drawing attention to the tests undertaken at three-month reviews (5.1) and always informing them of their results (2.6)  36. Sexual healthcare professionals should discuss the various ways that PrEP users could take PrEP in an unbiased manner (5.1) and engage in a shared decision-making process to decide whether switching regimens may be appropriate (9.2)  37. Sexual healthcare professionals should inform PrEP users how to access the sexual health service for ad-hoc adherence support between appointments and ensure contact details and opening hours are kept up to date on the sexual health service website (3.1) | 35. Modify – nice wording addressing side-effect management. Again, make sure materials are co-produced and that communication routes are acceptable to key populations. Could suggest a greater proportion of discussions cover side-effects which are very real for some people and do affect adherence. Amended and merged with 36  36. Modify – needs more focus on side-effects. Suggested wording ‘Sexual healthcare professionals should engage in well-informed and sophisticated discussions with PrEP users to create a personalised PrEP care plan”. Amended and merged with 35  37. Modify – don’t want to encourage people to have ad-hoc appointments but people do need the confidence to navigate the healthcare system (i.e. know they can come back to speak to a sexual healthcare professional, option to change regimens). Duplicate but tweaked so final rec includes specifics re: managing side-effects and is no longer a duplicate. New rec about a list of management strategies that can be shared with PrEP users | (PA7ii) PrEP providers should spend an adequate proportion of PrEP discussions educating PrEP users about possible side-effects and their typically transient nature and reassure against concerns about longer-term issues and create a personalised PrEP care plan, including information on switching regimens. *Reassurance can be provided by drawing attention to the regular reviews offered to PrEP users*  (PA7iv) PrEP providers and NGO staff (potentially through the use of peer navigators) should support PrEP users to navigate services and online information for appropriate expert support. *Support could include: ensuring PrEP users know they can return to or call the PrEP service to discuss side-effects and have the option to change regimens; and raising awareness of and directing PrEP users to reputable online sources of side-effect management*  (PA7i) PrEP services should provide PrEP providers and NGO staff with a list of management strategies for common side effects that they can share with PrEP users |
| PrEP users find it difficult to stay on PrEP for as long as relevant because their sexual partner(s) is suspicious of PrEP use as they associate it with promiscuity and infidelity | -- | “*I think he thought if I was on it, I was having sex with other people, and there was that...he kind of said, well, why would you be on it if we’re together now? And in my mind, I was thinking, well, I want to be on it just to make sure*.” (Stopped using PrEP) | Social influences  Skills | Enablement  Training | 13.2 Framing/ reframing  3.1 Social support (unspecified)  4.1 Instruction on how to perform the behaviour | 38. Ensure PrEP information and communications (e.g. national patient information booklet, posters in clinic waiting areas and consultation rooms and NGO settings, via sexual health services, NGO, and HIV/PrEP activists’ websites and social media, marketing campaign) address PrEP-related stigma, for example, by adopting ‘needs-based’ terminology rather than focusing on ‘risk’ and presenting PrEP as a responsible choice and positive means of reducing the likelihood of acquiring HIV, and include specific content on PrEP use within the context of a relationship to enable supportive and well-informed discussions among sexual partners (13.2)  30. Sexual healthcare professionals and NGO staff should encourage and support PrEP users to have wholistic conversations with their sexual partner(s) about the meaning of PrEP and the boundaries of the relationship (3.1), for instance, by sharing example phrases that they could incorporate into discussions (4.1) | 38. Modify – the first bit is not a priority here, will be addressed in stage 1. However, the last few lines are important and nice wording.  30. Modify – maintain the ethos of sexual healthcare professionals using their professional judgement to suggest/encourage and support PrEP users to have discussions with important others about PrEP and what it means in order to help people initiate (stage 2) and stay on PrEP though not necessarily something you do for everyone, tailor to the individual. Something quite novel to help support PrEP users stay on PrEP could be to recommend that sexual healthcare professionals explore and probe motivations for PrEP users wanting to stopping PrEP, including sexual partners’ reactions / views / perceptions | (PA7v) PrEP information and communications should include specific content on PrEP use within the context of relationships to address PrEP stigma, enable supportive and well-informed discussions among sexual partners, and prevent discontinuation of PrEP where there is an ongoing identified need. *Ensure that materials are co-produced and that communication routes are acceptable to key populations*  (PA7iii) PrEP providers and NGO staff should consider sexual partners’ reactions, views, and perceptions when exploring and probing PrEP users’ motivations for wanting to stop or having stopped using PrEP, be cognisant of sexual partner influences on PrEP users’ decisions to remain on PrEP, and use their professional judgement to encourage and support PrEP users to have wholistic conversations with their sexual partner(s) about the meaning of PrEP and boundaries of the relationship(s). *Share co-produced example phrases that PrEP users could incorporate into discussions* |
| PrEP users find it difficult to stay on PrEP for as long as relevant because they acquire recurrent STIs while on PrEP | -- | “*PrEP coming around allowed them [patients] to feel that they could have more sex, with different people, and not use condoms, and not have to sit and panic about it either. And some of them have come back subsequently and said, oh I'm getting other infections, I don't like this anymore, I'm going back to using condoms*.” (Sexual healthcare professional) | Beliefs about consequences | Education  Persuasion | 5.1 Information about health consequences  13.2 Framing/ reframing | 28. Sexual healthcare professionals and NGO staff should advise PrEP users that PrEP only protects against HIV and present the likelihood of contracting an STI following non-condom protected intercourse, taking account of various risk factors (5.1)  29. Sexual healthcare professionals and NGO staff should encourage PrEP users to continue using condoms alongside PrEP by framing PrEP as an additional rather than alternative HIV prevention method (13.2) | 28. Reject – already in national guidelines.  29. Reject – there is some dissonance / a PrEP paradox here as the majority of users are eligible because of condomless sex… so if they start using condoms then they may no longer be eligible. Condoms should be mentioned as part of combination prevention or, for the majority of PrEP users, as an option for STI prevention. But framing it as additional HIV prevention could undermine the message about the effectiveness of PrEP | -- |
|  | PrEP users find it easy to stay on PrEP for as long as relevant because of the positive health, emotional, and social consequences of PrEP (e.g. effectively safeguards their own and other people’s sexual health, reassures against personal HIV fear, social acceptability) | “*I don’t see that [stopping PrEP] being something I would consider in the short to medium term. I just feel that it gives me reassurance, both in terms of medical reassurance but also psychological reassurance*.” (PrEP user) | Beliefs about consequences | Education  Persuasion | 5.1 Information about health consequences  5.3 Information about social and environmental consequences  5.6 Information about emotional consequences | 02. PrEP information and communications (e.g. national patient information booklet, posters in clinic waiting areas and consultation rooms and NGO settings, via sexual health services, NGO, and HIV/PrEP activists’ websites and social media, marketing campaign) should include education on the positive health impacts of PrEP, as well as the wider social and emotional benefits and value of PrEP, for communities and individuals (5.1, 5.3, 5.6) | 02. Modify – lose the details of examples of types of information and communications | (PA7vi) PrEP information and communications should include education on the positive health impacts of PrEP, as well as the wider social and emotional benefits and value of PrEP, for communities and individuals |
