# Supplementary material for: Improving HIV pre-exposure prophylaxis (PrEP) adherence and retention in care: Process evaluation and recommendation development from a nationally implemented PrEP programme
Source: PLoS One. 2023 Oct 9;18(10):e0292289. doi: 10.1371/journal.pone.0292289 (PMC10561843; doi:10.1371/journal.pone.0292289)
Supplement: S8 Table — (DOCX) [file pone.0292289.s008.docx]

**S8 Table. Priority area 8 - A BCW analysis of ‘PrEP providers communicate the decision to not provide further PrEP’**

| **Barriers** | **Facilitators** | **Indicative quotes** | **TDF domains** | **Intervention Functions** | **Potential BCTs**  from the BCTTv1 (Michie et al. 2013) | **Initial recommendations for those considering implementing PrEP at scale**  Numbers in brackets = BCTs | **Post-APEASE and expert input decision**  Accept/Reject/Modify | **Agreed final recommendations** **for those considering implementing PrEP at scale** |
| --- | --- | --- | --- | --- | --- | --- | --- | --- |
| PrEP providers find it difficult to communicate the decision to not provide further PrEP because of inadequate discussion with PrEP users about the risk-benefit of PrEP at the outset, owing to a lack of knowledge, skills, and experience | PrEP providers find it easy to communicate the decision to not provide further PrEP because they mention at the start that need for PrEP may change over time (i.e. circumstance-dependent) and that ongoing eligibility will be assessed and is required to keep issuing PrEP | “*It becomes an issue when there are some reasons maybe not to give PrEP, there are some side-effects, or there's some effect on renal function. And then having to go back and talk about the risk-benefits again. In lots of people, that tends to be not fully discussed properly, it’s kind of glossed over*.” (Sexual healthcare professional)  “*We advise that there will be continuing assessments of their eligibility and it may be that they will drop in and out of eligibility depending on their sexual risk because we understand that that changes with time for everybody. So, we do make it very clear that that will happen*.” (Sexual healthcare professional) | Knowledge  Skills  Professional role and identity | Education  Training  Persuasion  Modelling | 5.1 Information about health consequences  4.1 Instructions on how to perform the behaviour  7.1 Prompts/cues  6.1 Demonstration of the behaviour  8.1 Behavioural practice/rehearsal  2.2 Feedback on behaviour  5.3 Information about social and environmental consequences  9.1 Credible source | 20. Ensure sexual healthcare professionals are educated, trained, and appraised in their skills in discussing the risks and benefits of PrEP (e.g. through online modules, peer support, clinical supervision), for example, by giving information on PrEP health consequences (5.1), producing a ‘how to’ script for common PrEP scenarios based on the lessons learned of sexual healthcare professionals with general medicine expertise (4.1, 7.1), and providing opportunities to shadow (6.1), practice with (8.1), and receive feedback (2.2) from more experienced sexual healthcare professionals  15. Mandate discussion with PrEP users about the risk-benefit of PrEP in a formal protocol that details the key activities required to be completed by sexual healthcare professionals at the initial PrEP assessment (4.1, 5.1)  21. Share positive testimonials (e.g. via emails, intranet) of respected senior sexual healthcare professionals promoting the social benefits of communicating the risk-benefit of PrEP, idea of ‘seasons of risk’, and requirement for checks on and ongoing need for PrEP at the initial PrEP consultation (5.3, 9.1) | 20. Modify – too generic in its current form, applies to all clinical care. Tighten to be more specific to PrEP e.g. training for sexual healthcare professionals to understand and explain instances in which stopping PrEP may be in the PrEP user’s best interests (think about the wording) and a UKMEC style PrEP document with examples of clear situations where the risk outweighs the benefits that sexual healthcare professionals can refer to *  15. Reject – already part of clinical governance/ competence. Worry that it separates PrEP from broader combination prevention. More important recommendations to prioritise  21. Reject – part of clinical governance / CPD | (PA8i) PrEP services should use multi-methods to develop PrEP providers’ knowledge of and skills in explaining instances when stopping PrEP may be in a PrEP user’s best interests. *For example, develop and educate PrEP providers on guidance that includes examples of situations where the risk of PrEP outweighs the benefits, co-produce scripts that address a range of literacy needs for common PrEP risk-benefit scenarios, and provide opportunities to shadow, practice, and receive feedback on communicating decisions to stop PrEP* |
