# Supplementary material for: Improving HIV pre-exposure prophylaxis (PrEP) adherence and retention in care: Process evaluation and recommendation development from a nationally implemented PrEP programme
Source: PLoS One. 2023 Oct 9;18(10):e0292289. doi: 10.1371/journal.pone.0292289 (PMC10561843; doi:10.1371/journal.pone.0292289)
Supplement: S9 Table — (DOCX) [file pone.0292289.s009.docx]

**S9 Table. Priority area 9 - A BCW analysis of ‘PrEP providers explore PrEP users’ reasons for wanting to stop/stopping using PrEP’**

| **Barriers** | **Facilitators** | **Indicative quotes** | **TDF domains** | **Intervention Functions** | **Potential BCTs**  from the BCTTv1 (Michie et al. 2013) | **Initial recommendations for those considering implementing PrEP at scale**  Numbers in brackets = BCTs | **Post-APEASE and expert input decision**  Accept/Reject/Modify | **Agreed final recommendations** **for those considering implementing PrEP at scale** |
| --- | --- | --- | --- | --- | --- | --- | --- | --- |
| PrEP providers find it difficult to explore PrEP users’ reasons for wanting to stop / stopping using PrEP because PrEP users tend not to discuss their thoughts about stopping PrEP / decision to stop PrEP before stopping | PrEP providers find it easy to explore PrEP users’ reasons for wanting to stop / stopping using PrEP because there are follow-up and/or other targeted intervention processes in place | “*Generally, we wouldn’t see them again, they just don't access the service, because obviously they feel they don't need it at the moment. So, they don't need PrEP, and they’ve not been for a sexual health screen. But if they do come back for a sexual health screen, then we'd say, I see you’ve dropped your PrEP, why was that. And kind of just reflect on it with them, is that the decision that they're happy with, and do they still want to remain off PrEP*.” (Sexual healthcare professional) | Behavioural regulation  Environmental context and resources | Education  Enablement  Environmental restructuring | 5.1 Information about health consequences  9.1 Credible source  1.8 Behavioural contract  1.9 Commitment  1.1 Goal setting (behaviour)  7.1 Prompts/cues  12.2 Restructure the social environment | 31b. Use a multi-method approach, including posters, national patient information booklets, positive testimonials of PrEP users, online resources, and verbal communication by sexual healthcare professionals and NGO staff, to emphasise the importance of PrEP users discussing stopping PrEP with a sexual healthcare professional (5.1, 9.1)  01. Sexual healthcare professionals could ask PrEP users to verbally agree to or sign a written contract specifying that they will attend for regular PrEP reviews, even if they have stopped PrEP in the interim period (1.8, 1.9, 1.1)  9. Integrate ‘pop-up’ messages into the IT system to alert sexual healthcare professionals that the patient presenting at their clinic did not attend or is overdue a PrEP appointment and advise them to raise the issue and explore if they have stopped using PrEP and if so, why (7.1)  10b. Run a monthly report on the IT system to identify ‘did not attends’ and those overdue a PrEP appointment and attempt to make contact (e.g. via email, phone, SMS) and discuss their decision to stop using PrEP, if applicable (12.2) | 31b Reject –emphasising the importance of discussing stopping PrEP with a sexual healthcare professional is already included as part of 31a. No point repeating so makes sense to include for PA3 ‘PrEP users attend PrEP reviews’ rather than for this PA in Table 2  01. Modify – impractical and too paternalistic in current form. However, duplicate and made more sense to include rec in priority area 3 ‘PrEP users attend PrEP reviews’ than here  9. Reject – favour automated system which sends reminders to PrEP users. Duplicate  10b. Accept – about knowing your PrEP cohort (at service-level) | (PA9i) PrEP services should assess monitoring and evaluation data to identify ‘did not attends’ and those overdue a PrEP review and attempt to make contact to discuss decisions to stop using PrEP and reengage them with PrEP care, as appropriate |
