# Supplementary material for: Improving HIV pre-exposure prophylaxis (PrEP) adherence and retention in care: Process evaluation and recommendation development from a nationally implemented PrEP programme
Source: PLoS One. 2023 Oct 9;18(10):e0292289. doi: 10.1371/journal.pone.0292289 (PMC10561843; doi:10.1371/journal.pone.0292289)
Supplement: S10 Table — (DOCX) [file pone.0292289.s010.docx]

**S10 Table. Priority area 10 - A BCW analysis of ‘PrEP users stop using PrEP’**

| **Barriers** | **Facilitators** | **Indicative quotes** | **TDF domains** | **Intervention Functions** | **Potential BCTs**  from the BCTTv1 (Michie et al. 2013) | **Initial recommendations for those considering implementing PrEP at scale**  Numbers in brackets = BCTs | **Post-APEASE and expert input decision**  Accept/Reject/Modify | **Agreed final recommendations** **for those considering implementing PrEP at scale** |
| --- | --- | --- | --- | --- | --- | --- | --- | --- |
| PrEP users find it difficult to stop using PrEP because of the social acceptability of PrEP and emerging stigmas around *not* using PrEP | -- | “*The decision to come off [PrEP] is much harder and more layered than deciding to go on it in the first place…I’ve got an option to continually be safe and have that faith in it, why would I ditch that faith in it. So, there’s guilt there personally. But, again with Grindr…it’s a bit like, well if I’m changing my setting to [HIV] negative instead of being on PrEP, what am I saying? Am I basically saying, one that I’m not valuing my own sexual health and two am I not valuing their sexual health*?” (Stopped using PrEP) | Beliefs about consequences | Education  Persuasion | 5.1 Information about health consequences  13.2 Framing/ reframing | 33. Use a range of educational methods to enhance PrEP users’ understanding of behaviours and situations that carry a higher likelihood of acquiring HIV and facilitate accurate assessments of when they no longer have a need for PrEP (5.1)  34. PrEP information and communications (e.g. interactions with sexual healthcare professionals and NGO staff, national patient information booklets, sexual health services, NGO, and HIV/PrEP activists’ websites and social media, marketing campaigns) should address emerging stigmas around people *not* using PrEP by framing it as an additional rather than alternative HIV prevention method (i.e. one of many options) (13.2) and sharing information on the effectiveness of alternative sexual health promotion methods that offer sufficient protection against HIV (5.1) | 33. Reject – more important to encourage people to start PrEP than to stop. It’s also incredibly difficult to assess risk for non-GBMSM PrEP users. Duplicate  34. Modify – the focus should be on the importance of offering choices and explaining the ‘seasons of risk’ concept because of emerging stigmas around *not* using PrEP. Inform people of all options for HIV prevention and ensure information and communications are tailored to the needs of distinct key populations (bearing in mind many of those who stand to benefit from PrEP may not be reached by current channels) | (PA10i) PrEP and wider sexual health resources and communications should inform of all options for HIV prevention, emphasise the importance of choices, and explain the ‘seasons of risk’ concept to address emerging stigmas around *not* using PrEP. *Ensure that materials are co-produced and that communication routes are acceptable to key populations* |
| -- | PrEP users find it easy to stop using PrEP because of a reduction in their self-perceived HIV risk (e.g. not planning any sexual activity, in a monogamous relationship) | “*We just got to the point in the relationship where we had a discussion about being exclusive, about sex, about safe sex and made a decision not to see anybody else, be monogamous, and I then took the decision to come off PrEP because I didn’t think I needed it anymore*.” (Stopped using PrEP) | Beliefs about consequences | Education  Persuasion | 5.1 Information about health consequences | 33. Use a range of educational methods to enhance PrEP users’ HIV literacy and ensure they have an accurate understanding of behaviours and situations that carry a higher likelihood of acquiring HIV (5.1) | 33. Reject – more important to encourage people to start PrEP than to stop. It’s also incredibly difficult to assess risk for non-GBMSM PrEP users. Duplicate | -- |
